# Combination of autoantibodies against NY-ESO-1 and viral capsid antigen immunoglobulin A for improved detection of nasopharyngeal carcinoma

**DOI:** 10.3892/ol.2014.2286

**Published:** 2014-06-25

**Authors:** YU-HUI PENG, YI-WEI XU, SI-QI QIU, CHAO-QUN HONG, TIAN-TIAN ZHAI, EN-MIN LI, LI-YAN XU

**Affiliations:** 1 Department of Clinical Laboratory, The Cancer Hospital of Shantou University Medical College, P.R. China; 2 The Key Laboratory of Molecular Biology for High Cancer Incidence Coastal Chaoshan Area, The Cancer Hospital of Shantou University Medical College, Shantou, Guangdong 515041, P.R. China; 3 Institute of Oncologic Pathology, Shantou University Medical College, Shantou, Guangdong 515041, P.R. China; 4 The Breast Center, The Cancer Hospital of Shantou University Medical College, Shantou, Guangdong 515041, P.R. China; 5 Department of Oncological Research Lab, The Cancer Hospital of Shantou University Medical College, Shantou, Guangdong 515041, P.R. China; 6 Department of Radiation Oncology, The Cancer Hospital of Shantou University Medical College, Shantou, Guangdong 515041, P.R. China; 7 Department of Biochemistry and Molecular Biology, Shantou University Medical College, Shantou, Guangdong 515041, P.R. China

**Keywords:** NY-ESO-1, autoantibody, nasopharyngeal carcinoma, viral capsid antigen immunoglobulin A, diagnosis

## Abstract

Nasopharyngeal carcinoma (NPC) is one of the most common malignant tumors in Southern China and Southeast Asia, and early detection remains a challenge. Autoantibodies have been found to precede the manifestations of symptomatic cancer by several months to years, making their identification of particular relevance for early detection. In the present study, the diagnostic value of serum autoantibodies against NY-ESO-1 in NPC patients was evaluated. The study included 112 patients with NPC and 138 normal controls. Serum levels of autoantibodies against NY-ESO-1 and classical Epstein-Barr virus marker, viral capsid antigen immunoglobulin A (VCA-IgA), were measured by enzyme-linked immunosorbent assay. Measurement of autoantibodies against NY-ESO-1 and VCA-IgA demonstrated a sensitivity/specificity of 42.9/94.9% [95% confidence interval (CI), 33.7–52.6/89.4–97.8%] and 55.4/95.7% (95% CI, 45.7–64.7/90.4–98.2%), respectively. The area under receiver operating characteristic curve for autoantibodies against NY-ESO-1 (0.821; 95% CI, 0.771–0.871) was marginally lower than that for VCA-IgA (0.860; 95% CI, 0.810–0.910) in NPC. The combination of autoantibodies against NY-ESO-1 and VCA-IgA yielded an enhanced sensitivity of 80.4% (95% CI, 71.6–87.0%) and a specificity of 90.6% (95% CI, 84.1–94.7%). Moreover, detection of autoantibodies against NY-ESO-1 could differentiate early-stage NPC patients from normal controls. Our results suggest that autoantibodies against NY-ESO-1 may serve as a potential biomarker, as a supplement to VCA-IgA, for the screening and diagnosis of NPC.

## Introduction

Nasopharyngeal carcinoma (NPC) is one of the most common cancers in Southern China and Southeast Asia, where the incidence rate ranges from 20 to 50 per 100,000 and is approximately 100-fold higher than that in the Western world (1,2). The five-year survival rate for patients with early-stage disease is ≤95%; however, for patients with stage III and IV disease, the five-year overall survival rate declines to ~70% (3,4). The majority of cases of NPC often present at an advanced stage at the time of diagnosis, due to its deep location and vague symptoms. Therefore, a screening protocol for the early diagnosis of NPC is urgently required, and may contribute to improving the treatment outcome.

Detection of Epstein-Barr virus (EBV) DNA and antibodies against EBV antigens, such as viral capsid antigen immunoglobulin A (VCA-IgA), is the method currently used for the serological diagnosis of NPC; however, specificity and sensitivity of these methods are considered unsatisfactory (5–9). A large number of studies describe the presence of autoantibodies to tumor-associated antigens (TAAs) in serum samples from patients with a variety of types of cancer, including NPC (10–15). Changes in the levels of gene expression and aberrant expression of tissue-restricted gene products are thought to mainly account for the humoral immune response to TAAs, which functions to remove precancerous lesions during the early events of carcinogenesis (16–19). Autoantibodies have been found to precede manifestations of symptomatic cancer, and detection of autoantibodies may be useful in types of cancer where there are high-risk populations (20). Thus, identification of novel autoantibody biomarkers may lead to early diagnosis or prediction of disease progression in patients with NPC.

Cancer-testis antigens (CTAs), a group of tumor antigens, are encoded by genes that are normally expressed only in the human germline, but are also expressed in various tumor types. CTAs are widely explored as both a diagnostic marker and a therapeutic target in malignant lesions (21). As one of the most immunogenic CTAs, the NY-ESO-1 antigen was originally found in esophageal cancer by serological recombinant cDNA expression cloning. The aberrant expression of NY-ESO-1 has been observed in a variety of neoplasms. NY-ESO-1 elicits both humoral and cellular immune responses in patients with NY-ESO-1-expressing tumors (22). It has been estimated that 10–50% of patients with NY-ESO-1-expressing tumors develop antibody responses (23). Autoantibodies against NY-ESO-1 present in a broad variety of cancer types, such as lung, breast and prostate cancer, providing a possibility of early detection (10,24,25). However, to the best of our knowledge, the potential value of autoantibodies against NY-ESO-1 as biomarker in the evaluation of NPC has not yet been studied.

Combined analysis of VCA-IgA and NY-ESO-1 autoantibodies may increase the ability to detect NPC. In the present study, the diagnostic value of serum autoantibodies against NY-ESO-1 in NPC were investigated, and the possible correlation between NY-ESO-1 autoantibodies and clinical parameters was explored. Additionally, assay of VCA-IgA, a serological marker in clinical use for NPC, was conducted.

## Materials and methods

### Study population

The study comprised 112 patients with NPC at the Department of Radiation Oncology, The Cancer Hospital of Shantou University Medical College (Shantou, China) between December 2012 and July 2013. NPC was defined on the basis of a routine diagnostic workup comprised of nasopharyngoscopy and radiological imaging techniques [computed tomography (CT), magnetic resonance imaging (MRI) and ultrasonography], and was biopsy-proven in all poorly differentiated squamous carcinoma types. Tumor stage was defined according to the seventh edition of the UICC/AJCC staging system for nasopharyngeal carcinoma (26). The control group consisted of 138 healthy volunteers with no previous malignant disease. The patient group and the control group were matched as closely as possible, in terms of age and gender (Table I).

Patients were all newly diagnosed. Peripheral blood samples from normal controls and NPC patients, obtained at the time of diagnosis and prior to any therapeutic procedures, were centrifuged at 1,250 × g for 5 min at room temperature, and stored at −80°C until use. Prior to the use of these clinical materials for investigation, approval for the study from the institutional ethics review committee of Shantou University Medical College and written informed consent from all patients were obtained. This study was conducted in accordance with the principles set out in the Declaration of Helsinki.

### NY-ESO-1 protein expression and purification

The full-length cDNA for NY-ESO-1 was cloned in the pDEST17 (Invitrogen Life Technologies, Carlsbad, CA, USA) expression vector. The resulting recombinant plasmids were verified by sequencing prior to expression trials. To obtain the recombinant proteins, the expression host *E. coli* Rosetta (DE3) (Novagen, Darmstadt, Germany) was transformed with the recombinant plasmid. Selection of transformed colonies was performed on LB-agar plates containing 100 μg/ml ampicillin [Sangon Biotech (Shanghai) Co., Ltd., Shanghai, China). Cells carrying the recombinant plasmid were inoculated in 5 ml LB medium supplemented with 100 μg/ml ampicillin, and cultured overnight at 37°C with rotary shaking (~250 × g). Subsequently, the cell culture was transferred to 2 l of fresh LB medium supplemented with 100 μg/ml ampicillin. When the optical density (OD) at 600 nm reached 0.4–0.6, IPTG (Merck, Darmstadt, Germany) was added to a final concentration of 0.4 mM to induce the expression of recombinant protein. The cells were grown at 30°C and, after ~3 h, were harvested by centrifugation at 14,800 × g for 10 min at 4°C. The bacterial pellet was resuspended in 1X phosphate-buffered saline (PBS; 2.7 mM KCl, 4.3 mM Na_2_HPO_4_, 1.8 mM KH_2_PO_4_, 137 mM NaCl; pH 7.4) buffer supplemented with 8 M urea and 1 mM phenylmethylsulfonyl fluoride. The cell fractions were purified under denaturing conditions and were refolded *in vitro* while immobilized on a Ni^2+^-NTA-Sepharose (Novagen) column. Following incubation, the column was washed with wash buffer A (1X PBS, 8 M urea; pH 8.0) followed by wash buffer B (1X PBS). The refolded proteins were eluted with elution buffer (PBS, 500 mM imidazole; pH 7.4) and dialyzed twice against 4 l of 50% glycerol in PBS. Protein concentrations were determined by bicinchoninic acid (BCA) protein assay, using the BCA Protein Assay kit (Thermo Scientific, Pierce Biotechnology, Rockford, IL, USA), and bovine serum albumin (BSA; Thermo Fisher Scientific, Boston, MA, USA) was used as a standard. The purity of the recombinant protein was assessed by Coomassie Blue staining (Thermo Fisher Scientific), following SDS-PAGE.

### Measurement of serum autoantibodies against NY-ESO-1

Autoantibodies against NY-ESO-1 were measured by enzyme-linked immunosorbent assay (ELISA). Purified recombinant antigen, NY-ESO-1, was diluted in 50 mM bicarbonate buffer (pH 9.6) to a final concentration of 0.1 μg/ml. The antigen dilutions were dispensed into 96-well microtiter plates (100 μl/well; Haotian Biotechnology Co., Ltd., Haimen, China) and incubated overnight at 4°C. The plates were washed three times with phosphate-buffered saline containing 0.05% Tween 20 [PBST; Sangon Biotech (Shanghai) Co., Ltd.], and then blocked with a blocking buffer (PBST containing 1% BSA) at 37°C for 1 h, followed by three washes with PBST. Serum samples and quality control samples (QCS, a pooled plasma sample collected randomly from 50 patients with NPC), were diluted 1/110 in blocking buffer, then were incubated at 37°C for 1 h, as were appropriate control rabbit polyclonal anti-human NY-ESO-1 antibodies (Immunosoft, Zhoushan, China) specific for capture proteins. After washing four times with PBST, polyclonal horseradish peroxidase (HRP)-conjugated goat anti-human IgG or anti-rabbit IgG (Santa Cruz Biotechnology, Inc., Santa Cruz, CA, USA) were used as secondary antibodies at the dilution recommended by the manufacturer. After a 60-min incubation, the plates were washed, and pre-prepared 3,3′,5,5′-tetramethylbenzidine (Intec Products, Inc., Xiamen, China) and hydrogen peroxide (Intec Products, Inc.) were added. Color formation was allowed to proceed for 15 min, before termination with 0.5 M H_2_SO_4_. The absorbance of each well was read at 450 nm and referenced to 630 nm by a MK3 microplate reader (Thermo Fisher Scientific).

All cancer and normal samples were interspersed on the plates and run in duplicate. QCSs were run to ensure quality control monitoring of the assay runs by using Levey-Jennings plots. With the purpose of minimizing an intra-assay deviation, the ratio of the difference between duplicated sample OD values to their sum was used to assess the precision of the assay. If the ratio was >10%, the test of this sample was considered invalid and the sample was repeated.

### ELISA for EBV VCA-IgA

Concentrations of VCA-IgA in all samples were determined in duplicate by ELISA using the EB virus VCA-IgA detection kit (Berer Bioengineering, Beijing, China). The experiments were conducted according to the manufacturer’s instructions. Briefly, one well of blank control, two wells of negative controls and two wells of positive controls were included on each plate, then each serum sample was added to the plates (100 μl in each well) at a 1:10 dilution and allowed to incubate at 37°C for 30 min. After washing five times, 100 μl of HRP-conjugated anti-human IgA antibody was added into each well. Following incubation at 37°C for 30 min, the plates were then washed. Color development was conducted by the addition of 100 μl tetramethylbenzidine substrate solution and incubation at 37°C for 15 min. When the reaction had stopped, absorbance of test sera was immediately read on the microplate reader (Thermo Fisher Scientific), using 450 nm as the primary wavelength and 630 nm as the secondary wavelength.

### Statistical analysis

All analyses were performed using SPSS, version 17.0 (SPSS, Inc., Chicago, IL, USA) or GraphPad Prism software (GraphPad Software, Inc., La Jolla, CA, USA). The number and proportion of positive samples are presented with the exact 95% confidence interval (CI) for binomial proportions (27). The comparison of NY-ESO-1 autoantibody and VCA-IgA between sera of normal controls and NPC was conducted by the use of the nonparametric Mann-Whitney U test. The cutoff value for the VCA-IgA assay was calculated according to the manufacturer’s recommendations. Receiver operating characteristic (ROC) curve analysis was performed to determine the cutoff value of the autoantibody assay. In addition, an area under the ROC curve (AUC) with 95% CI was calculated for both NY-ESO-1 autoantibody and VCA-IgA. χ^2^ tests were carried out to identify correlations between the positivity of the different markers and clinical parameters. In all tests, two-sided P<0.05 was considered to indicate a statistically significant difference.

## Results

### Serum levels of autoantibodies against NY-ESO-1 and VCA-IgA between NPC patients and normal controls

**Figure 1:**
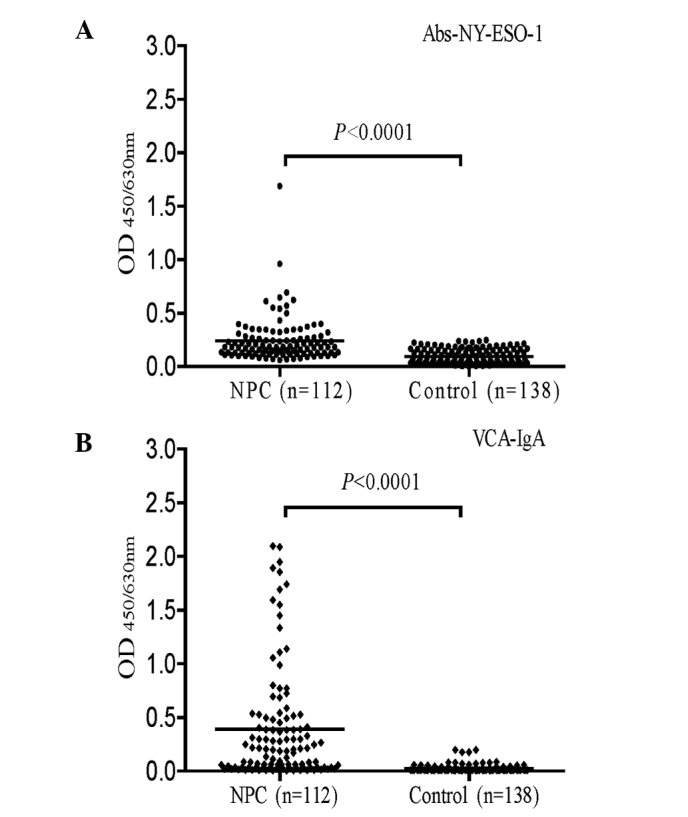
Scatter plots of OD values at a wavelength of 450/630 nm. OD values of individual NPC patients and normal controls (A) for autoantibodies against NY-ESO-1 and (B) for VCA-IgA. Statistical significance was determined by means of Mann-Whitney U test. Black horizontal lines are the means. OD, optical density; NPC, nasopharyngeal carcinoma; VCA-IgA, viral capsid antigen immunoglobulin A; Abs, autoantibodies.

The mean OD_450/630_ ± standard deviation of serum autoantibodies against NY-ESO-1 was 0.241±0.206 in the 112 NPC patients and 0.095±0.067 in the 138 normal controls. The serum levels of autoantibodies against NY-ESO-1 were significantly higher in the NPC patients than in the normal controls (P<0.0001; Fig. 1A). The levels of VCA-IgA were also significantly higher in patients with NPC than in the normal controls (0.390±0.524 versus 0.024±0.033; P<0.0001; Fig. 1B).

### Evaluation of autoantibodies against NY-ESO-1 as diagnostic marker

**Figure 2:**
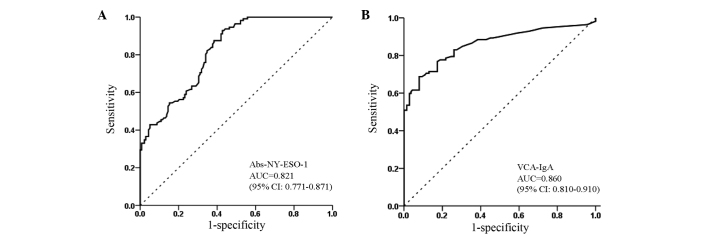
ROC curve analysis. An AUC value with 95% CI was calculated for both (A) NY-ESO-1 autoantibody and (B) VCA-IgA. ROC, receiver operating characteristic; AUC, area under the ROC curve; VCA-IgA, viral capsid antigen immunoglobulin A; Abs, autoantibodies.

The diagnostic value of autoantibodies against NY-ESO-1 was evaluated using the ROC analysis. According to the ROC curve, the optimal cutoff value of autoantibodies against NY-ESO-1 for NPC was 0.210, providing a sensitivity of 42.9% (95% CI, 33.7–52.6%) and a specificity of 94.9% (95% CI, 89.4–97.8%) (Table II). ROC curve analysis showed that VCA-IgA alone (AUC, 0.860; 95% CI, 0.810–0.910; Fig. 2B) was marginally more effective than autoantibodies alone against NY-ESO-1 (AUC=0.819, 95% CI, 0.770–0.869; Fig. 2A). According to the manufacturer’s instructions, the recommended clinical cutoff value of VCA-IgA was 0.150. The sensitivity and specificity of VCA-IgA were 55.4% (95% CI, 45.7–64.7%) and 95.7% (95% CI, 90.4–98.2%), respectively. The efficacy of combination of autoantibodies against NY-ESO-1 and VCA-IgA is presented in Table II. Use of the combination of autoantibodies against NY-ESO-1 and VCA-IgA provided an enhanced sensitivity of 80.4% (95% CI, 71.6–87.0%) and a specificity of 90.6% (95% CI, 84.1–94.7%). Predictive values and likelihood ratios for autoantibodies against NY-ESO-1, VCA-IgA and both markers combined in the diagnosis of NPC are shown in Table II. These results indicate that NY-ESO-1 autoantibodies may be a potentially useful serum biomarker for NPC, particularly when used in conjunction with VCA-IgA.

In this study, 23 patients with NPC had early-stage disease (AJCC stage I and II). The positive rates of autoantibodies against NY-ESO-1 and VCA-IgA in NPC patients with early-stage disease were 47.8% (95% CI, 27.4–68.9%) and 39.1% (95% CI, 20.5–61.2%), respectively, which were significantly higher than those in the normal controls (P<0.0001; Table III). This indicates that detection of autoantibodies against NY-ESO-1 enables discrimination between early-stage NPC and normal controls.

### Correlations between positive rates of autoantibodies against NY-ESO-1, VCA-IgA and clinicopathological variables

Analysis of serum samples in NPC patients showed that autoantibodies against NY-ESO-1 did not significantly differ with age, gender, T stage, N stage or overall stage (Table IV). The correlations of VCA-IgA and both markers combined with clinicopathological characteristics in NPC patients were also evaluated. However, there was no correlation with any of the variables (Table IV).

## Discussion

NY-ESO-1 was discovered based on its capacity to incite a humoral immune response in cancer patients. The elevated levels of the serum autoantibodies against NY-ESO-1 have been reported in various types of cancer (10,24,25). The present study demonstrated that the detection of autoantibodies against NY-ESO-1 in the peripheral blood has potential diagnostic value for NPC. The AUC for autoantibodies against NY-ESO-1 in NPC patients was 0.821 (95% CI, 0.771–0.871), with a sensitivity of 42.9% (95% CI, 33.7–52.6%) and specificity of 94.9% (95% CI, 89.4–97.8%). The diagnostic accuracy of autoantibodies against NY-ESO-1 in NPC was greater than those previously detected in breast cancer, lung cancer and neuroblastoma (10,11,24,28,29).

Detection of NPC patients at the early stage is essential for optimal prognosis (3,4). A promising approach for the early detection of cancer is to measure circulating antibodies to TAAs in patient serum other than markers from cancer cells (19). Previous investigations showed that autoantibodies can be detectable as early as five years before radiographic detection on incidence screening in lung cancer, and can be detected in the asymptomatic stage of breast cancer up to five years before the onset of disease (30,31). Our study also indicates that the induction of autoantibodies against NY-ESO-1 occurs early in the process of carcinogenesis, which is consistent with previous studies on other types of cancer (10,24,25,32). A higher proportion of patients with early-stage NPC had positive results for autoantibodies against NY-ESO-1 than for VCA-IgA (Table III). Although statistical analysis showed that the correlation of positivity of autoantibodies against NY-ESO-1 was not significant between early- and advanced-stage patients, there appeared to be a higher incidence of autoantibodies in patients with early-stage tumors than in the advanced disease group. These results indicate that autoantibodies against NY-ESO-1 may be a promising marker for the early detection of NPC. However, the number of the NPC patients with early-stage disease in this study was relatively small. Thus, it is necessary to further validate these results using a large cohort of early-stage NPC patient samples.

In the current study, VCA-IgA was demonstrated to be a useful diagnostic biomarker of NPC, which is in agreement with a study by Chang *et al* (33). However, this assay used alone is not sensitive enough for the purpose of primary screening. Autoantibodies against NY-ESO-1 have a marginally lower AUC value than the VCA-IgA marker; however, it is a molecular marker rather than a traditional EBV marker for diagnosis of NPC. Notably, the combined use of autoantibodies against NY-ESO-1 with a classical EBV marker increased its diagnostic sensitivity (Table II). As a screening test, high sensitivity should be a prerequisite in order that potential patients are not missed. In this case, to increase the sensitivity and marginally reduce the specificity as a tradeoff can be justified. The combination of autoantibodies against NY-ESO-1 and VCA-IgA resulted in a high sensitivity of 80.4% (95% CI, 71.6–87.0%), while maintaining a specificity of 90.6% (95% CI, 84.1–94.7%), which demonstrated greater diagnostic efficacy compared with the classic VCA-IgA test alone (Table II). These results suggest autoantibodies against NY-ESO-1 may be a good supplement to VCA-IgA for NPC primary screening.

This study is the first to suggest that autoantibodies against NY-ESO-1 may represent a potential non-EBV serum marker for the diagnosis of NPC, particularly in early-stage NPC patients. Moreover, the results revealed that the combined detection of autoantibodies against NY-ESO-1 and VCA-IgA could increase the sensitivity with modest specificity for the serological screening and diagnosis of NPC.

## Figures and Tables

**Table I:** Characteristics of the study population.

	NPC patients	Normal controls
Number	112	138
Gender		
Male	86	90
Female	26	48
Mean age ± SD (years)	49±10	50±9
Age range (years)	28–76	40–71
T stage		
T1	11	-
T2	36	-
T3	35	-
T4	30	-
N stage		
N0	11	-
N1	41	-
N2	53	-
N3	7	-
M stage		
M0	108	-
M1	4	-
Overall stage		
I	1	-
II	22	-
III	53	-
IV	36	-

NPC, nasopharyngeal carcinoma; T, tumor; N, node; M, metastasis.

**Table II:** Results for measurement of Abs-NY-ESO-1, VCA-IgA and both markers combined in the diagnosis of NPC.

NPC vs. NC	Abs-NY-ESO-1	VCA-IgA	Abs-NY-ESO-1 + VCA-IgA
Sensitivity (%)	42.9 (33.7–52.6)	55.4 (45.7–64.7)	80.4 (71.6–87.0)
Specificity (%)	94.9 (89.4–97.8)	95.7 (90.4–98.2)	90.6 (84.1–94.7)
PPV (%)	87.3 (74.9–94.3)	91.2 (81.1–96.4)	87.4 (79.0–92.0)
NPV (%)	67.2 (60.0–73.6)	72.5 (65.3–78.7)	85.0 (78.0–90.2)
Positive LR	8.45 (3.98–17.94)	12.72 (5.72–28.34)	8.53 (5.04–14.43)
Negative LR	0.60 (0.51–0.71)	0.47 (0.38–0.57)	0.22 (0.15–0.32)

Values in brackets represent the 95% CI. Abs, autoantibodies; VCA-IgA, viral capsid antigen immunoglobulin A; NPC, nasopharyngeal carcinoma; NC, normal control; PPV, positive predictive value; NPV, negative predictive value; LR, likelihood ratio.

**Table III:** Positive rates of Abs-NY-ESO-1, VCA-IgA and both markers combined between early stage NPC and normal controls.

		Abs-NY-ESO-1		VCA-IgA		Abs-NY-ESO-1+VCA-IgA	
				
	n	Positive (%, 95% CI)	P-value	Positive (%, 95% CI)	P-value	Positive (%, 95% CI)	P-value
Early-stage (I+II)	23	11 (47.8, 27.4–68.9)	P<0.0001	9 (39.1, 20.5–61.2)	P<0.0001	17 (73.9, 51.3–88.9)	P<0.0001
Normal	138	7 (5.1, 2.2–10.6)		6 (4.3, 1.8–9.6)		13 (9.4, 5.3–15.9)	

Statistical significance was determined by the χ^2^ test. Abs, autoantibodies; VCA-IgA, viral capsid antigen immunoglobulin A; NPC, nasopharyngeal carcinoma; CI, confidence interval.

**Table IV:** Association of positive rates of Abs-NY-ESO-1, VCA-IgA and both markers combined with clinicopathologic characteristics in NPC patients.

		Abs-NY-ESO-1		VCA-IgA		Abs-NY-ESO-1+VCA-IgA	
				
	n	Positive (%, 95% CI)	P-value	Positive (%, 95% CI)	P-value	Positive (%, 95% CI)	P-value
Gender							
Male	86	36 (41.9, 31.5–53.0)	0.698	49 (57.0, 45.9–67.5)	0.531	69 (80.2, 70.0–87.7)	0.952
Female	26	12 (46.2, 27.1–66.3)		13 (50.0, 30.4–69.6)		21 (80.8, 60.0–92.7)	
Age, years							
≤50	53	23 (43.4, 30.1–57.6)	0.913	28 (52.8, 38.8–66.5)	0.610	40 (75.5, 61.4–85.8)	0.217
>50	59	25 (42.4, 29.8–55.9)		34 (57.6, 44.1–70.2)		50 (84.7, 72.5–92.4)	
T stage							
T1+T2	47	22 (46.8, 32.4–61.8)	0.472	21 (44.7, 30.5–59.8)	0.053	36 (76.6, 61.6–87.2)	0.394
T3+T4	65	26 (40.0, 28.3–52.9)		41 (63.1, 50.2–74.4)		54 (83.1, 71.3–90.9)	
N stage							
N0+N1	52	26 (50.0, 36.0–64.0)	0.155	24 (46.2, 32.5–60.4)	0.068	40 (76.9, 62.8–87.0)	0.394
N2+N3	60	22 (36.7, 24.9–50.2)		38 (63.3, 49.8–75.1)		50 (83.3, 71.0–91.3)	
Overall stage							
I+II (early-stage)	23	11 (47.8, 27.4–68.9)	0.589	9 (39.1, 20.5–61.2)	0.079	17 (73.9, 51.3–88.9)	0.563
III+IV(advanced-stage)	89	37 (41.6, 31.2–52.5)		53 (59.6, 48.6–69.7)		73 (82.0, 72.1–89.1)	

Statistical significance was determined by the χ^2^ test. Abs, autoantibodies; VCA-IgA, viral capsid antigen immunoglobulin A; NPC, nasopharyngeal carcinoma; CI, confidence interval.
